# Early Implementation of the Navigator-Assisted Hypofractionation (NAVAH) Program in Hispanic-American Breast Cancer Patients

**DOI:** 10.7759/cureus.83727

**Published:** 2025-05-08

**Authors:** Shearwood McClelland III, Abizairie Sanchez-Feliciano, Nelly Davila, Tamika K Smith, Ursula J Burnette, Louisa Onyewadume, Chesley W Cheatham

**Affiliations:** 1 Radiation Oncology, University of Oklahoma Health Sciences Center, Oklahoma City, USA; 2 Radiation Oncology, University Hospitals Seidman Cancer Center, Case Western Reserve University School of Medicine, Cleveland, USA; 3 Radiation Oncology, El Centro de Servicios Sociales, Lorain, USA; 4 Radiation Oncology, Case Western Reserve University School of Medicine, Cleveland, USA; 5 Radiation Oncology, Duke University Medical Center, Durham, USA

**Keywords:** breast cancer, financial toxicity, hispanic ethnicity, patient navigation, radiation therapy

## Abstract

Although cancer-related mortality in the United States (US) has generally been decreasing, this has not occurred among Hispanic populations, where cancer is the leading cause of death. The population growth of the Hispanic population in the US from 2010 to 2021 was substantially larger than the overall US population growth rate (24% vs. 7%). This increased population growth adds importance to addressing cancer care delivery barriers facing this patient population. We discuss early implementation of the Navigator-Assisted Hypofractionation (NAVAH) program in addressing these barriers.

## Editorial

Although cancer-related mortality in the United States (US) has generally been decreasing, this has not occurred among populations of Hispanic ethnicity, where cancer is the leading cause of death [1,2]. The population growth of the Hispanic population in the US from 2010 to 2021 increased by 24%, substantially larger than the overall US population growth rate of 7% [3]. Consequently, addressing barriers to optimal delivery of cancer care will only gain importance as the population of Hispanic-Americans increases [4].

Radiation therapy (RT) is a mainstay of cancer care, significantly improving outcomes across multiple disease sites. The US Hispanic population faces multiple barriers to optimal RT, including socioeconomic status, language barriers to preventative care, migration status, and higher rates of uninsured populations [4]. The initial analysis of disparities in access to radiation oncology care facing Hispanic-American populations occurred in 2017, which revealed that Hispanic-Americans were less likely to receive any RT (including definitive RT) in certain disease types such as breast cancer, prostate cancer, and head and neck cancer [5]. A 2024 update of this analysis revealed Hispanic patients have more than twice the gross number of cancer cases diagnosed under age 50 compared with White patients, present at a more advanced cancer stage, and suffer a median 9-day delay in starting RT compared with non-Hispanic White patients [4,6]. Furthermore, US Hispanic cancer patients compared with non-Hispanic Whites are more commonly uninsured, receive RT less often regardless of insurance status, are less likely to receive invitations for clinical trial participation, have a greater distance to travel to receive RT, and are less likely to travel far distances for RT [7-11].

Among Hispanic-American women, the most common cancer is breast cancer; previous work has revealed that Hispanic patients are less likely to receive breast conservation therapy (lumpectomy + adjuvant RT) and much less likely to complete RT as prescribed [12,13]. Compared with White patients, Hispanic patients have lower odds of timely completion of short-course (1-4 week) or conventional (5-7 week) RT; shorter course RT may reduce care (treatment completion) disparities facing Hispanic breast cancer patients [14,15]. Furthermore, short-course RT may alleviate the disproportionate financial toxicity suffered by Hispanic patients; previous work has indicated that six percent of all Hispanic-Americans with early-stage breast cancer will lose their homes due to the cumulative financial toxicity of cancer treatment [16].

The application of patient navigation to increase access to short-course RT is a novel approach in combating RT access disparities, comprising the basis for the Navigator-Assisted Hypofractionation (NAVAH) program, which began in breast cancer patients of African-American race and has been expanded to African-American prostate cancer patients [17,18]. This report represents the early stages of NAVAH expansion to breast cancer patients of self-reported Hispanic ethnicity via our ongoing Phase I trial (Clinical Trials NCT05978232 [21]) in partnership with a local community organization (El Centro de Servicios Sociales, Inc.). Details regarding survey administration have been previously reported [19].

Findings thus far from our first three patients indicate that our previously validated Spanish-language surveys [20] are feasible in the Hispanic breast cancer population. Previously established survey categories of acceptability (comfort and bias in system interactions), accessibility (transportation, distance, healthcare literacy), accommodation (internet access, transportation navigation), affordability (financial concerns, employment, education), and availability (care access and coordination) [20] have thus far from initial results revealed evidence of good availability, excellent accessibility, good affordability, outstanding accommodation, average acceptability, and good knowledge of cancer screening and treatment. Objective assessment of financial toxicity via the validated Comprehensive Score for Financial Toxicity-Functional Assessment of Chronic Illness Therapy (COST-FACIT) has been feasible to date, with data gathered at pre-RT and one-month post-RT intervals to date, with plans to collect six-month post-RT data to complete financial toxicity analysis.

Conclusion

This report represents evidence that the implementation of the NAVAH program is feasible in the Hispanic breast cancer population. As this Phase I trial continues to accrue, we expect additional information to guide our ability to optimize the impact of patient navigation to help meet the logistic and financial toxicity challenges commonly faced by this patient population.
